# Real-world therapeutic strategies in active adult-onset Still’s disease: clinical insights from the GIRRCS-AOSD study group

**DOI:** 10.3389/fimmu.2026.1852661

**Published:** 2026-08-05

**Authors:** Piero Ruscitti, Daniela Iacono, Flavia Riccio, Mariachiara Visconti, Antonio Vitale, Valeria Caggiano, Jessica Sbalchiero, Ludovico De Stefano, Clelia Zampaglione, Lidia La Barbera, Luca Navarini, Fabiola Atzeni, Francesco Ursini, Francesco Caso, Luisa Costa, Marcella Prete, Federico Perosa, Giuliana Guggino, Serena Bugatti, Carlomaurizio Montecucco, Claudia Fabiani, Luca Cantarini, Paola Cipriani, Francesco Ciccia, Roberto Giacomelli

**Affiliations:** 1 Department of Biotechnological and Applied Clinical Sciences, University of L’Aquila, L’Aquila, Italy; 2 Department of Precision Medicine, University of Campania “Luigi Vanvitelli”, Naples, Italy; 3 Rheumatology Unit, Department of Medical Sciences, Surgery and Neurosciences, University of Siena and Azienda Ospedaliero-Universitaria Senese [European Reference Network (ERN) for Rare Immunodeficiency, Autoinflammatory, and Autoimmune Diseases (RITA) Center], Siena, Italy; 4 Department of Internal Medicine and Therapeutics, University of Pavia, Pavia, Italy; 5 Division of Rheumatology, Fondazione IRCCS Policlinico San Matteo, Pavia, Italy; 6 Department of Health Promotion, Mother and Child Care, Internal Medicine and Medical Specialties, Rheumatology Section-”P. Giaccone”, University of Palermo, Palermo, Italy; 7 Clinical and Research Section of Rheumatology and Clinical Immunology, Fondazione Policlinico Campus Bio-Medico, Via Álvaro del Portillo, Rome, Italy; 8 Rheumatology and Clinical Immunology, Department of Medicine, School of Medicine, University of Rome "Campus Biomedico", Rome, Italy; 9 Rheumatology Unit, Department of Experimental and Internal Medicine, University of Messina, Messina, Italy; 10 Medicine and Rheumatology Unit, IRCCS Istituto Ortopedico Rizzoli, Bologna, Italy; 11 Department of Biomedical and Neuromotor Sciences, Alma Mater Studiorum University of Bologna, Bologna, Italy; 12 Rheumatology Unit, Department of Clinical Medicine and Surgery, University of Naples Federico II, Naples, Italy; 13 Internal Medicine Unit, Department of InterdisciplinaryMedicine, University of Bari Medical School, Bari, Italy; 14 Rheumatic and Systemic Autoimmune Diseases Unit, Department of Interdisciplinary Medicine (DIM), University of Bari Medical School, Bari, Italy; 15 Ophthalmology Unit, Department of Medicine, Surgery and Neurosciences, University of Siena, Siena, Italy

**Keywords:** adult onset Still’s disease, IL-1 inhibitor, IL-6 inhibitor, Still’s disease, therapy

## Abstract

**Objectives:**

To evaluate the therapeutic management of patients with active adult-onset Still’s disease (AOSD) in a multicentre real-world setting and to provide and compare current real-world treatment strategies with existing recent international recommendations.

**Methods:**

From January 2022 to December 2023, 173 consecutive patients with active AOSD attending centres participating in the *Gruppo Italiano di Ricerca in Reumatologia Clinica e Sperimentale* (GIRRCS) AOSD research study group were prospectively enrolled. Demographic, clinical, and laboratory data were collected, and therapeutic strategies prescribed by the treating physicians were systematically recorded. Factors associated with the administration of biologic disease-modifying antirheumatic drugs (bDMARDs), including IL-1 and IL-6 inhibitors, were analysed.

**Results:**

Glucocorticoids were administered in 97.7% of patients. Conventional synthetic DMARDs were prescribed in 58.8% of cases, mainly methotrexate and cyclosporin A. Overall, 43.3% of patients received bDMARDs, predominantly IL-1 and IL-6 inhibitors. Specifically, 33.5% were treated with IL-1 inhibitors, 5.8% with IL-6 inhibitors, and 4.0% with tumour necrosis factor inhibitors. After three months of treatment, 86.7% of patients achieved clinical inactive disease as assessed by the treating physician. Comparing recorded treatment strategies with international recommendations, no compliance was found regarding the use of glucocorticoids, which are recommended to be markedly limited or avoided, and early administration of bDMARDs, which were administered within 3 months in a minority of patients.

**Conclusions:**

This multicentre real-world study outlines current therapeutic approaches for patients with active AOSD and highlights a gap between international treatment recommendations and clinical practice, underscoring the need for further studies to optimize patient management.

## Introduction

Adult onset Still’s disease (AOSD) is a rare systemic inflammatory disorder of unknown aetiology (1). It is currently recognised as an inflammatory disease located at the intersection between autoinflammatory and autoimmune disorders (2). Indeed, both innate and adaptive immune pathways are implicated in driving a dysregulated inflammatory response and disease development (2). Clinically, AOSD is classically characterised by daily spiking fever, arthritis, and an evanescent salmon-coloured rash, frequently associated with marked hyperferritinaemia (3). Nonetheless, the clinical expression of the disease is highly heterogeneous, ranging from relatively mild presentations to severe, life-threatening conditions (4). In a subset of patients, prominent multi-organ involvement and serious complications may occur, substantially complicating disease management and adversely affecting long-term prognosis (4, 5).Based on the underlying inflammatory pathogenic mechanisms (1–3), immunosuppressive therapies represent the cornerstone of treatment in AOSD. Therapeutic strategies commonly include glucocorticoids (GCs), conventional synthetic disease-modifying antirheumatic drugs (csDMARDs), and biologic DMARDs (bDMARDs) (6, 7). GCs are generally considered first-line therapy and should be tapered as soon as clinically feasible (6). When disease activity is insufficiently controlled or GC dependence develops, csDMARDs, most frequently methotrexate, are introduced as second-line agents (6). In patients considered refractory or intolerant of first- and second-line treatments, bDMARDs, particularly interleukin (IL)-1 and IL-6 inhibitors, are commonly prescribed, with accumulating evidence supporting their sustained efficacy and safety (7, 8). More recently, innovative therapeutic approaches have proposed the early use of bDMARDs to enhance clinical response rates and increase the likelihood of drug-free remission (9). Accordingly, the latest European Alliance of Associations for Rheumatology (EULAR)/Paediatric Rheumatology European Society (PReS) recommendations advocate prioritising bDMARDs to maximise remission achievement (10). However, a well-defined therapeutic algorithm remains lacking, especially in adult patients, and clinical practice often relies on empirical decision-making.

On this basis, we aimed to evaluate the therapeutic management of patients with active AOSD in a multicentre real-world study, providing a comprehensive overview of current clinical practice and exploring its concordance with recent EULAR/PReS recommendations. Additionally, with exploratory purposes, we assessed possible predictive factors associated with the use of IL-1 and IL-6 inhibitors to better define clinical profiles related with the administration of these drugs in our clinical practice by physicians in charge for patient management.

## Patients and methods

From January 2022 to December 2023, consecutive patients with active AOSD attending centres participating in the *Gruppo Italiano di Ricerca in Reumatologia Clinica e Sperimentale* (GIRRCS) AOSD research study group were prospectively evaluated at baseline and after three months of follow-up. All patients fulfilled the Yamaguchi classification criteria for AOSD (11). Patients with other inflammatory diseases, malignancies, or active infections were excluded (4, 5). Specifically, potential infectious causes were excluded through blood cultures, serological testing, nasal swabs, PCR analyses, chest X-rays, and abdominal ultrasonography. In cases of persistent suspicion despite these evaluations, computed tomography and/or positron emission tomography-CT were incorporated into the diagnostic workup, as previously described (4, 5).

The GIRRCS AOSD research cohort is an Italian multicentre study involving Rheumatology Units across Italy. A specific study framework was designed to derive criteria for the assessment of disease activity, focusing on changes in clinical variables following therapeutic interventions rather than on direct evaluation of drug efficacy (12). In addition to its primary objectives, the cohort provided the opportunity to investigate real-world therapeutic strategies for AOSD and to explore the applicability of recent EULAR/PReS recommendations in routine clinical practice. The systematic literature review underpinning the EULAR/PReS recommendations covered the period from 2013 to October 2022, overlapping with our enrolment period and therefore reflecting the evidence available during that timeframe (10). We considered this comparison informative because it could provide some insights into the extent to which emerging evidence had already been translated into routine clinical practice before the formal publication of these recommendations. Therefore, our aim was to assess the degree of concordance between real-world management strategies and the principles that were subsequently formalized by the EULAR/PReS task force (10). To evaluate the possible concordance of current clinical practice from recent EULAR/PReS recommendations, we translated the narrative statements of recommendations into assessed parameters, focusing on therapeutic strategies and defining three levels of concordance: i. “full concordance,” when items were met; ii. “partial concordance,” when items were partly fulfilled; iii. “no concordance,” when items were not satisfied.

For the present analysis, only patients with active disease at baseline and a prospective follow-up of three months were included (12). Disease status was rated as “active” or “clinically inactive” according to the treating physician’s judgement. Clinically inactive disease was defined as the absence of clinical signs and symptoms together with normalization of inflammatory markers for at least two consecutive months, irrespective of ongoing therapy. The definition of clinically inactive disease relied on anamnestic information collected by the treating physicians within the context of routine clinical practice. Active disease was defined by the persistence of clinical manifestations or by the need for additional or intensified treatment to control disease activity (12). Considering the present evaluation, the inclusion criterion was the presence of active disease, whereas patients with clinically inactive disease were excluded among those diagnosed with AOSD according to the Yamaguchi criteria (11, 12). The following clinical and laboratory variables were systematically assessed at baseline and after three months: fever, skin rash, arthralgia, arthritis, splenomegaly, lymphadenopathies, pleuritis, pericarditis, erythrocyte sedimentation rate (ESR), C-reactive protein (CRP), ferritin, and patient global assessment (PGA) of disease activity measured on a 10-cm visual analogue scale (VAS). Fever was defined as a body temperature ≥38.5 °C lasting from at least three days up to one week or longer (12). All selected variables were identified following a systematic literature review, expert opinion, and dedicated consensus discussions on disease activity in AOSD (13–15). Therapeutic regimens (ie, GCs, csDMARDs, and bDMARDs) were recorded. Furthermore, they were categorised as follows: low-to-medium GC dosage (≤0.5 mg/kg/day of prednisone equivalent), high GC dosage (>0.5 mg/kg/day of prednisone equivalent), GC plus csDMARD(s), GC plus bDMARD with or without csDMARD(s), and csDMARD(s) plus bDMARD(s), as previously described (16, 17). Treatment decisions were not protocol-driven, consistent with the real-world nature of the study.

The study was approved by the Ethics Committee of ASL1 Avezzano-Sulmona-L’Aquila (L’Aquila, Italy; Ref. Nos. 0139815/16 and 0095184/20) and conducted in accordance with Good Clinical Practice and the Declaration of Helsinki. Written informed consent was obtained from all participants. Data handling complied with EU General Data Protection Regulation (GDPR) requirements (Regulation 2016/679/EU).

### Statistical analysis

Statistics were used to outline real-world therapeutic practices in patients with active AOSD. Categorical variables were expressed as number and percentage, while continuous variables were reported as median and interquartile range with first and third quartiles (IQR, Q1-Q3), according to data distribution. Pre- and post-treatment comparisons were performed by using McNemar test for categorical variables and Wilcoxon rank test for continuous variables. Regression models were built to exploratorily identify predictors of IL-1 and IL-6 inhibitor administration, including age, male sex, CRP (assessed as continuous variable and dichotomized to ≥68.7 mg/L), and ferritin (assessed as continuous variable and dichotomized to ≥1225.0 ng/mL). These laboratory features were selected as markers of disease severity based on previous study explicitly deriving thresholds in this specific context of AOSD (18). These variables were included in our models primarily reflecting clinically relevant outcomes in patients with AOSD in terms of prognosis, namely macrophage activation syndrome and mortality (18), rather than as indicators of disease activity or treatment stratification. Given the aim of the regression analyses as exploratory, we sought to investigate whether markers of disease severity might be associated with the use of IL-1 or IL-6 inhibitors in clinical practice. Considering the number of events related to the administration of IL-6, only exploratory univariate analyses were performed. No patients had missing data at baseline and three months. A two-sided p value <0.05 was considered statistically significant. All analyses were performed using SPSS for Windows, version 22.0 (SPSS Inc., Chicago, IL, USA).

## Results

**Figure 1:**
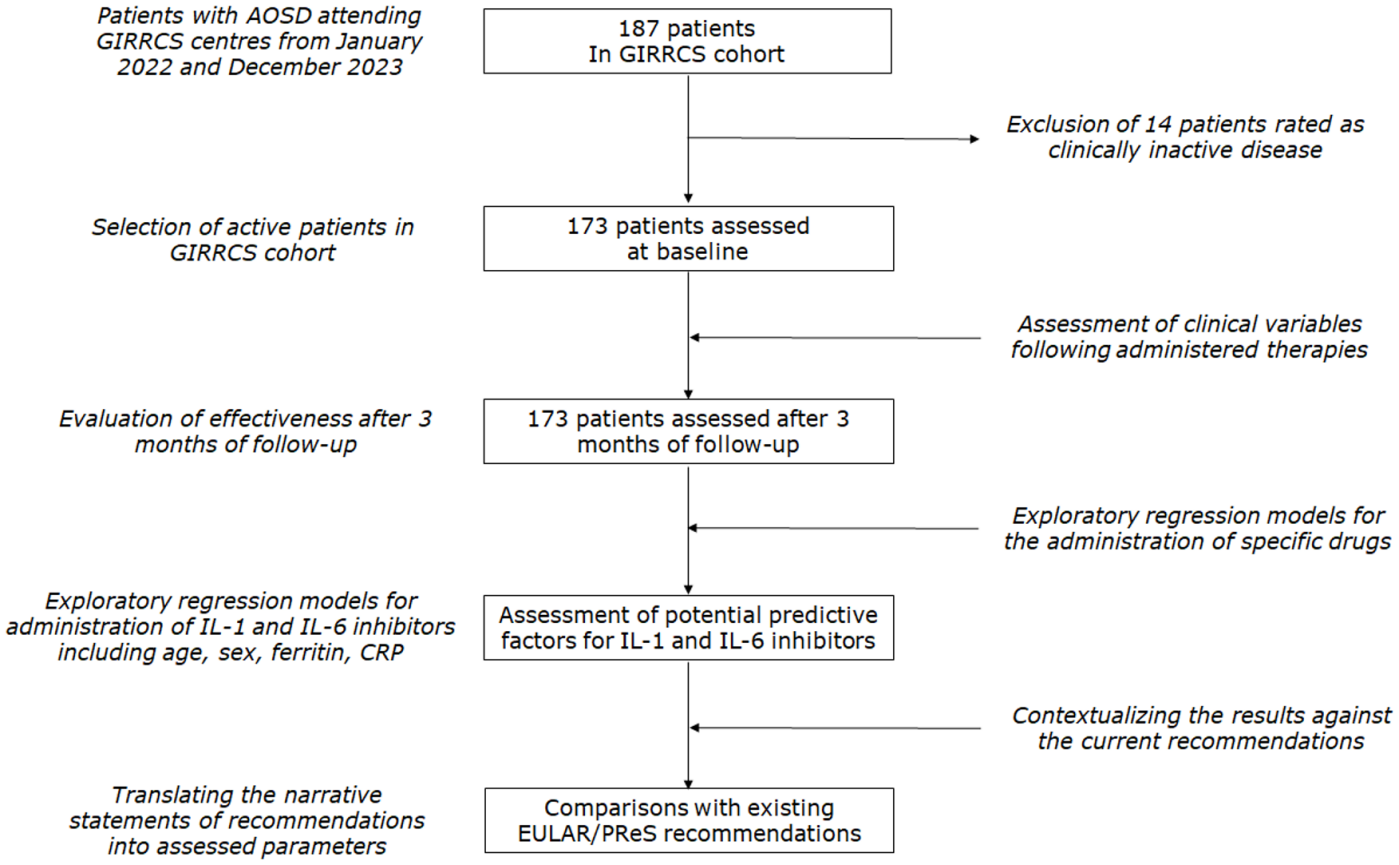
In this figure the flow-chart of the study is reported. From January 2022 to December 2023, 187 consecutive patients with AOSD attending centres participating in the GIRRCS-AOSD research study group were evaluated. After that, 173 active patients were selected for the purposes of the present study and evaluated at baseline and after three months of follow-up. Subsequently, potential predictive factors for the administration of both IL-1 and IL-6 inhibitors were exploratory evaluated. Finally, a comparison with existing recommendations was performed.

In our cohort, 187 patients with AOSD were assessed; 173 active patients (mean age 40.0 ± 17.1 years, male sex 50.3%) were evaluated in the present study whereas 14 clinical inactive patients were not analysed. Almost all these active patients were characterized by fever (93.1%) whereas skin rash and arthritis were recognized in 68.8% and in 56.1%, respectively. A study flow-chart is reported in **Figure 1**. Inflammatory markers were markedly increased; ESR 60.0 mm/hr (69.0, 28.0-97.0), CRP 46.6 mg/L (110.0, 12.0-122.0), and ferritin 1002.0 ng/ml (3702.0, 280.0-3982.0) were recorded in these patients. In this cohort, 42.2% of patients showed a CRP ≥ 68.7 mg/L whereas 45.1% ferritin ≥ 1225.0 ng/ml, respectively. Other descriptive findings are reported in **Table 1**.

**Table 1:** Clinical descriptive characteristics of assessed patients with AOSD at baseline.

Descriptive patient features	173 assessed patients
Male sex (%)	87 (50.3)
Age, years, mean ± SD	40.0 ± 17.1
Clinical characteristics	
Fever (%)	161 (93.1)
Arthralgia (%)	147 (85.0)
Skin rash (%)	119 (68.8)
Arthritis (%)	97 (56.1)
Lymphadenopathy (%)	93 (53.8)
Splenomegaly (%)	55 (31.8)
Pericarditis (%)	30 (17.3)
Pleuritis (%)	21 (12.1)
Patient global assessment, median (IQR, Q1-Q3)	7.0 (6.0, 4.0-10.0)
Laboratory findings	
ESR, mm/h, median (IQR, Q1-Q3)	60.0 (69.0, 28.0-97.0)
CRP, mg/L, median (IQR, Q1-Q3)	41.0 (112.0, 12.0-122.0)
CRP ≥68.7 mg/L (%)	73 (42.2)
Ferritin, ng/ml, median (IQR, Q1-Q3)	1002.0 (3702.0, 280.0-3982.0)
Ferritin ≥1225.0 ng/ml (%)	78 (45.1)
Therapies	
GCs (%)	169 (97.7)
High dose of GCs (%)	63 (36.4)
csDMARDs (%)	101 (58.8)
Methotrexate (%)	76 (43.9)
Cyclosporin (%)	15 (8.6)
Other csDMARDs (%)	10 (5.7)
Colchicine (%)	7 (4.0)
Time to bDMARD initiation, months median (IQR, Q1-Q3)	6.0 (10.0, 1.0-11.0)
bDMARDs (%)	75 (43.3)
IL-1 inhibitors (%)	58 (33.5)
IL-6 inhibitor (%)	10 (5.8)
TNF inhibitors (%)	7 (4.0)
GC monotherapy (%)	39 (22.5)
GCs+csDMARDs (%)	63 (36.4)
GCs+bDMARDs (%)	33 (19.1)
csDMARDs+bDMARDs (%)	4 (2.3)
GCs+csDMARDs+bDMARDs (%)	34 (19.7)
Outcome	
Clinical inactive disease after 3 months of treatment (%)	150 (86.7)

SD, standard deviation; IQR, Q1-Q3, range interquartile with first and third quartiles; ESR, erythrocyte sedimentation rate; CRP, C reactive protein; GCs, glucocorticoids; csDMARDs, conventional synthetic disease modifying anti rheumatic drugs; bDMARDs, biologic disease modifying antirheumatic drugs; IL, interleukin.

Glucocorticoids (GCs) were the most frequently administered treatment, being prescribed to 97.7% of patients, of whom 36.4% received high-dose therapy. Among other drugs, csDMARDs were given in 58.8% of patients, mainly methotrexate (MTX) and cyclosporin A. MTX (mean dosage 15 mg weekly) were administered in 43.9% of patients whereas cyclosporin A in 8.6% (5mg/kg daily). Concerning bDMARDs, 43.3% of patients were treated with such drugs, mostly IL-1 and IL-6 inhibitors. Specifically, 33.5% of patients were treated with IL-1 inhibitors (anakinra 100 mg daily or canakinumab 300 mg monthly), 5.8% with anti-IL-6 inhibitor (tocilizumab, 8 mg/kg monthly), 4.0% with TNF inhibitors (adalimumab 40 mg every other week, etanercept 50 mg weekly, infliximab 3 mg/kg every other month), respectively. The median time of bDMARD initiation was 6.0 months (10.0, 1.0-11.0); 22.7% of patients started a bDMARD within the first 3 months. Assessing the combination therapeutic strategies, GCs+csDMARD was recorded in 36.4% of patients, GCs+bDMARD in 19.1%, and GCs+csDMARD+bDMARD in 19.7%, respectively. Differently, a low percentage of patients 2.3% were treated with the combination therapy with csDMARD+bDMARD without the administration of GCs.

**Figure 2:**
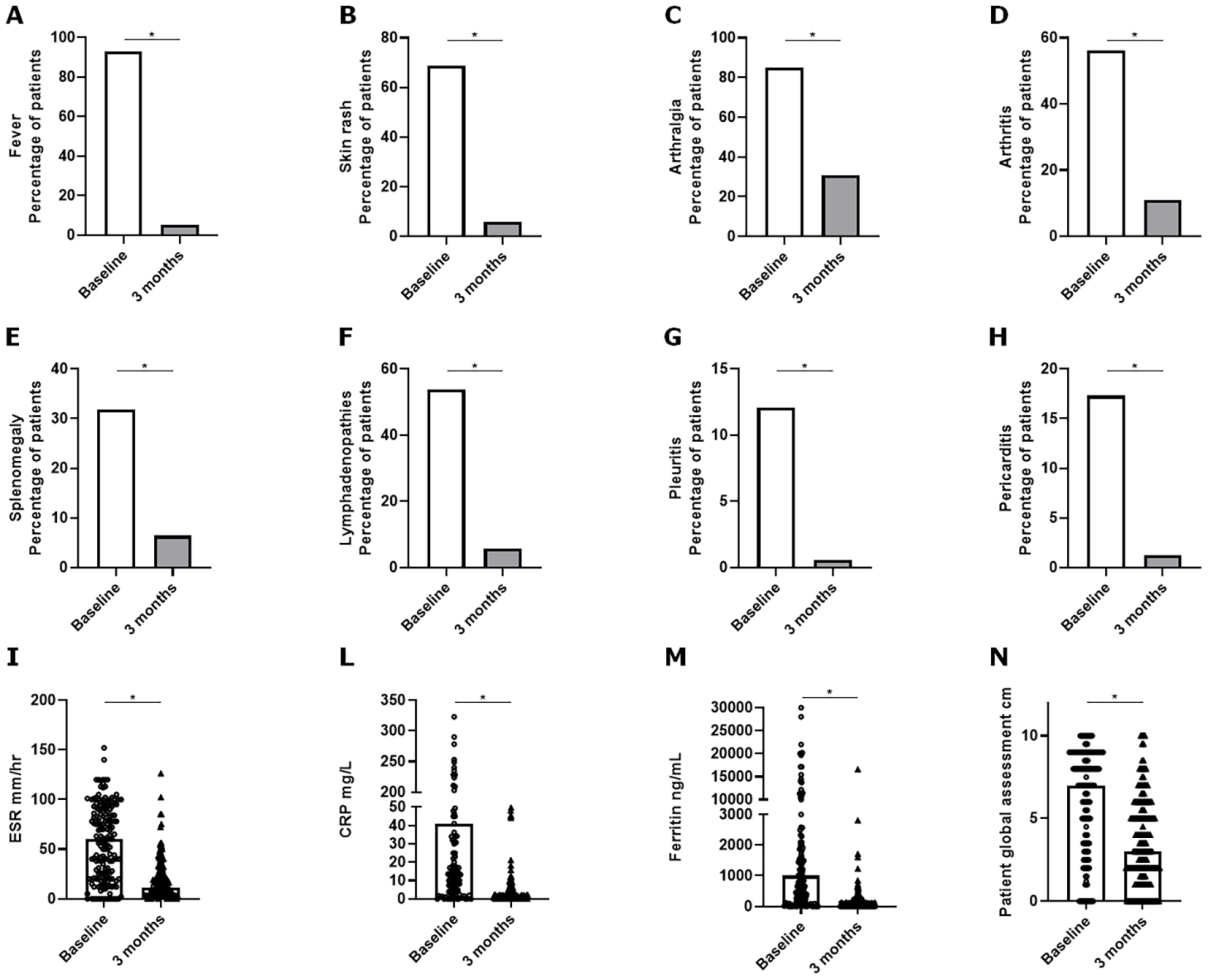
In this figure, the effectiveness on assessed clinical features is reported by pre/post comparisons which results all statistically significant (* p<0.05). Panel **(A)**, fever: baseline 93.1% vs 3 months 5.2%; Panel **(B)**, skin rash: baseline 68.8% vs 3 months: 5.8%; Panel **(C)**, arthralgia: baseline 85.0% vs 3 months: 30.6%; Panel **(D)**, arthritis: baseline 56.1% vs 3 months: 11.0%; Panel **(E)**, splenomegaly: baseline 31.8% vs 3 months: 6.4%; Panel **(F)**, lymphadenopathies: baseline 53.8% vs 3 months: 5.8%; Panel **(G)**, pleuritis: baseline 12.1% vs 3 months: 0.6%; Panel **(H)**, pericarditis: baseline 17.3% vs 3 months: 1.2%; Panel **(I)**, erythrocyte sedimentation rate (ESR) mm/h: baseline 60.0 (69.0, 28.0-97.0) vs 3 months: 11 (23.0, 4.0-27.0); Panel **(L)**, C reactive protein (CRP) mg/L: baseline 41.0 (112.0, 12.0-122.0) vs 3 months: 1.8 (5.4, 0.5-5.9); Panel **(M)** – ferritin ng/mL: baseline 1002.0 (3702.0, 280.0-3982.0) vs 3 months: 79.5 (180.6, 24.0-204.6); Panel **(N)** – patient global assessment cm (0-10): baseline 7.0 (6.0, 4.0-10.0) vs 3 months: 3 (3.8, 1.5-5.3).

After three months, 86.7% achieved the clinical inactive disease according to the physician in charge for the management of patients. This feature parallelled with a significant improvement of disease manifestations and laboratory markers, as reported in **Figure 2**. We did not stratify the results on effectiveness according to different therapeutic strategies, as this analysis was beyond the main scope of the present study. No changes in bDMARD therapy were observed during the relatively short follow-up period assessed in this study.

Exploiting predictive factors for the administration of IL-1 or IL-6 inhibitors with exploratory purposes, regression models were preliminarily built including into the model for age, male sex, CRP, and ferritin. These models were performed to provide a clinically tentative risk profile for the administration of these bDMARDs in real-world clinical practice by physicians in charge for patient management. In the multivariate exploratory model for the use of IL-1 inhibitors, the ferritin ≥ 1225.0 ng/ml resulted to be a significant predictor (OR: 2.81, 95%CI: 1.36-5.81, p=0.005) whereas age, male sex, and CRP were not associated. Assessing the predictive factors for the administration of IL-6 inhibitor by using preliminary univariate analyses, the age was significantly associated the administration of this drug (OR: 1.03, 95%CI: 1.01-1.07, p=0.046), whereas, male sex, CRP, and ferritin were not associated. These data are summarized in **Table 2** and they should be considered as exploratory.

**Table 2:** Exploratory regression analyses exploiting possible predictive factors for the administration of both IL-1 or IL-6 inhibitors in assessed patients with AOSD.

Clinical variables	OR	95% CI	P-value
Administration of IL-1 inhibitor			
Univariate analyses			
Age years	0.99	0.97-1.02	0.314
Male sex	1.09	0.58-2.05	0.789
CRP mg/L	0.99	0.98-1.01	0.078
CRP ≥68.7 mg/L	1.34	0.45-1.62	0.631
Ferritin ng/ml	1.01	0.96-1.04	0.088
Ferritin ≥1225.0 ng/ml	3.01	1.87-7.20	**0.004**
Multivariate analysis with CRP and Ferritin used as continuous variables			
Age years	0.99	0.97-1.09	0.056
Male sex	1.06	0.28-2.45	0.872
CRP mg/L	0.99	0.97-1.01	0.434
Ferritin ng/ml	1.01	0.98-1.02	0.073
Multivariate analysis with CRP≥68.7 mg/L and Ferritin≥1225.0 ng/ml			
Age years	0.99	0.97-1.01	0.533
Male sex	1.06	0.55-2.04	0.857
CRP ≥68.7 mg/L	1.18	0.58-2.40	0.657
Ferritin ≥1225.0 ng/ml	2.81	1.36-5.81	**0.005**
Administration of IL-6 inhibitor			
Univariate analyses			
Age years	1.03	1.01-1.07	**0.046**
Male sex	1.09	0.58-2.05	0.789
CRP mg/L	0.98	0.96-1.01	0.068
CRP ≥68.7 mg/L	0.99	0.98-1.02	0.065
Ferritin ng/ml	0.87	0.44-3.09	0.985
Ferritin ≥1225.0 ng/ml	0.99	0.97-1.01	0.098

OR, odds ratio; CI, confidence interval; CRP, C reactive protein.

Statistically significant values are shown in bold.

Taking all these findings together, we evaluated the concordance between real-world clinical practice observed in our cohort and the recent EULAR/PReS recommendations about treatment (10). Notably, no concordance was found regarding the use of GCs, which are recommended to be markedly limited or avoided (10). In fact, GCs were prescribed in 97.7% of patients in our cohort. Partial concordance was observed for the use of csDMARDs, which were administered in 58.8% of patients, despite the scarce supporting evidence (10). Finally, with respect to the early initiation of IL-1 and IL-6 inhibitors (10), the median time to bDMARD initiation in our cohort was 6 months, with only 22.7% of patients receiving these therapies within the first 3 months, highlighting no concordance with the recent EULAR/PReS recommendations. These findings are reported in **Table 3**.

**Table 3:** Comparison between the findings of the present study and the latest EULAR/PReS recommendations.

The EULAR/PReS recommendations about treatment*	Main items of the recommendations	Our findings	Compliance between recommendation items and our findings
To avoid prolonged systemic GC use for achieving and maintaining the target, the use of IL-1 and IL-6 inhibitors should be prioritised due to high evidence of efficacy.	GC use can be markedly limited, or even avoided.	97.7% treated with GCs.	No concordance in our real-world data.
	Evidences supporting the use of cs DMARDs is scarce.	58.8% treated with csDMARDs.	Partial concordance in our real-world data.
An IL-1 or an IL-6 inhibitor should be initiated as early as possible when the diagnosis is established.	IL-1 and IL-6 inhibitor should be initiated before 3 months from symptoms onset.	6 months to initiate a bDMARD; 22.7% started a bDMARD within the first 3 months.	No concordance in our real-world data.

*Fautrel B, et al. Ann Rheum Dis. 2024;83:1614-1627.

## Discussion

This multicentre real-world study may provide an overview of current therapeutic approaches to patients with active AOSD and highlights a gap between real-world clinical practice and the most recent EULAR/PReS treatment recommendations. Despite evolving evidence and guideline updates advocating early targeted therapy, management strategies in daily practice continue to rely predominantly on a step-up approach centred on GCs possibly delaying the administration of bDMARDs.

In our cohort, GCs, alone or in combination with csDMARDs and/or bDMARDs, remained the cornerstone of treatment, despite their well-recognised risk of dependency and cumulative toxicity, reported in up to 45% of patients (6). This finding may reflect longstanding clinical practice, as GCs are highly effective in rapidly controlling systemic inflammation and inducing initial clinical responses in most patients with AOSD (16). However, prolonged GC exposure is associated with predictable adverse effects, reinforcing the need for more GC-sparing strategies. Furthermore, csDMARDs, particularly MTX, were still widely prescribed, consistent with their traditional use in patients without severe systemic involvement or life-threatening complications (19). Nevertheless, 43.3% of patients required bDMARDs, with IL-1 and IL-6 inhibitors being the most frequently used agents, typically after failure of first- and second-line therapies. Anakinra and canakinumab, both licensed in Europe for AOSD, and tocilizumab, widely used off-label, have demonstrated robust efficacy and long-term safety in both clinical trials and real-world studies (20–22).

Despite this evidence, our data may suggest that bDMARDs are often introduced late in the disease course. In fact, the time of bDMARD initiation was 6 months and 22.7% of patients started the bDMARD within the first 3 months. This delayed initiation may compromise optimal disease control, as accumulating evidence suggests that early bDMARD administration improves clinical response rates, reduces GC exposure, and may prevent chronic disease evolution (23–25). In fact, an early targeted intervention during a proposed “window of opportunity” could suppress the acute inflammatory cascade, promote immunological remission, and minimise irreversible damage (23, 24). This paradigm shift is explicitly supported by recent EULAR/PReS recommendations, which emphasise early prioritisation of bDMARDs (10). Nonetheless, our findings may highlight that such recommendations are not yet fully implemented in daily practice with bDMARDs frequently reserved as a last-line therapeutic option (25). Several factors may contribute to this delay. Diagnostic challenges, particularly in patients presenting with fever of unknown origin, often lead to empirical GCs and antibiotic use before the proper recognition of AOSD. Another relevant factor may be the persistence of clinician hesitancy, particularly when evidence is derived from relatively limited datasets and observational studies, as is often the case in rare diseases. In these settings, physicians may continue to rely on personal experience, local practices, or established treatment algorithms, leading to a slower adoption of newly issued recommendations. Additionally, therapeutic strategies historically adapted from other rheumatic diseases, such as rheumatoid arthritis, may not fully address the unique clinical behaviour and outcomes of AOSD. Disease heterogeneity and rarity further complicate the adoption of uniform treatment algorithms, especially in adult populations where specific guidelines have only recently become available. In fact, differences in patient characteristics, disease severity, and previous treatment exposure may also contribute to deviations from recommendation-based management in real-world clinical practice.

Our analysis also may provide some exploratory and preliminary insights into potential predictive factors associated with the administration of bDMARDs in clinical practice. IL-1 inhibitors were more frequently administered in patients with a more pronounced hyperferritinaemia, a recognised marker of disease severity and adverse prognosis (18, 26). Increasing age was associated with IL-6 inhibitor use, likely reflecting therapeutic sequencing after failure of other interventions or specific safety and tolerability considerations in older patients (27, 28). Given the exploratory nature of these regression analyses, the findings should be interpreted with caution and considered hypothesis-generating, providing a basis for future specifically designed and adequately powered investigations.

Beyond clinical considerations, inequitable access to healthcare resources may represent a substantial barrier to early treatment with bDMARDs. While patients in well-resourced healthcare systems may benefit from early diagnosis and timely access to targeted therapies, others, particularly in lower-income settings, may experience delayed diagnosis and prolonged reliance on GCs and csDMARDs due to reimbursement restrictions and high costs of bDMARDs (29–31). Across Europe, access to bDMARDs for inflammatory arthritis varies widely, with some countries reporting that only 15-60% of eligible patients receive reimbursed bDMARDs (32–34). Such disparities may significantly affect outcomes and likely extend to rare diseases such as AOSD, underscoring the need for coordinated international efforts to improve equitable access to effective therapies. Thus, differences in healthcare systems and reimbursement policies may substantially influence treatment availability and prescribing patterns across countries and centres, limiting the practical implementation of recommended therapeutic strategies. In addition, access to bDMARDs may be restricted by regulatory requirements, administrative barriers, or funding constraints, resulting in therapeutic choices that do not fully align with current treatment recommendations.

This study has several limitations. Its observational design was not intended to evaluate drug efficacy but rather to describe physicians’ therapeutic decisions in real-world clinical settings, inherently limiting causal inference (35). Treatment allocation was not controlled, and inter-centre variability may have introduced measurement bias. Furthermore, the definition of clinically inactive disease relied on anamnestic information collected by the treating physicians within the context of routine clinical practice. Despite this approach is inherently reflective of real-world patient management, it also implies that disease activity status was not derived from systematically scheduled, protocol-driven assessments. Instead, it was determined on clinically documented patient history. Consequently, intermediate evaluations were not consistently recorded, which may have limited the temporal resolution of disease activity assessment. However, this methodological choice has been made to mirror everyday clinical practice and unlikely it substantially altered the overall interpretation of the results. Concerning AOSD disease duration, these data were not collected. We included time to bDMARD initiation as a surrogate measure of disease duration. Diagnostic delay in AOSD remains poorly investigated and dedicated studies are needed. Furthermore, the single-country design of this assessment may restrict the generalizability of the findings across diverse national and regulatory contexts. In addition, the regression analyses focusing on IL-6 inhibitors were underpowered and should be considered exploratory. In fact, the limited sample size and the small number of patients receiving IL-6 inhibitors reduced the robustness and generalizability of these findings, thereby underscoring the need for additional confirmatory studies. In addition, considering the real-world nature of our study and the exploratory intent of the regression analyses, we did not perform sensitivity analyses. In fact, sensitivity analyses are typically confirmatory tools, whereas our objective was to identify broad patterns and generate new hypotheses of work rather than to validate definitive conclusions. Moreover, although widely accepted, the Yamaguchi criteria do not include genomic testing and may lead to misclassification of other autoinflammatory conditions as AOSD (11). Given ongoing advances in genetic diagnostics, incorporating genomic testing, after appropriate evaluation of fever of unknown origin, may improve diagnostic accuracy and therapeutic precision (36–40). Furthermore, a subgroup analysis, stratified according to the four previously identified clinically derived disease clusters (4), was not feasible, as the limited sample size did not provide sufficient statistical power to ensure reliable results. Consequently, such analyses fall beyond the scope of the present study and should be more appropriately addressed in dedicated investigations involving larger cohorts. Finally, our assessment did not include patients with life-threatening complications, highlighting the need for further studies to fully elucidate the real-world management of these patients, who are characterized by a poor prognosis (41–45).

In conclusion, this real-world multicentre study may delineate the current therapeutic landscape of active AOSD and may reveal a gap between clinical practice and contemporary EULAR/PReS recommendations. In fact, GC-centred step-up strategies remain still prevalent, often delaying the initiation of bDMARDs which represent the most effective disease-modifying treatments. These findings may highlight the need for further efforts to promote guideline dissemination in prioritising an early targeted therapy and optimising outcomes for patients with AOSD.

## Data Availability

The original contributions presented in the study are included in the article/supplementary material. Further inquiries can be directed to the corresponding author.
